# *Talaromyces Marneffei* infections in five human immunodeficiency virus-negative patients with the history of predation on wild bamboo rats (*Rhizomys* spp) - a case series

**DOI:** 10.1186/s12879-025-10713-z

**Published:** 2025-03-11

**Authors:** Liming Cao, Yilan Sun, Ziyuan Zhou, Xiaxia Pan

**Affiliations:** 1 Cancer Center, Department of Pulmonary and Critical Care Medicine, Zhejiang Provincial People’s Hospital (Affiliated People’s Hospital), Hangzhou Medical College, Hangzhou, 310014 Zhejiang China; 2 State Key Laboratory for Diagnosis and Treatment of Infectious Diseases, Collaborative Innovation Center for Diagnosis and Treatment of Infectious Diseases, The First Affiliated Hospital, National Clinical Research Center for Infectious Diseases, Zhejiang University School of Medicine, Hangzhou, 310003 China

**Keywords:** *Talaromyces Marneffei*, Talaromycosis, Wild animals, Bamboo rats

## Abstract

**Background:**

Talaromycosis is an aggressive and life-threatening disease, caused by the pathogen *Talaromyces marneffei* (*T. marneffei*) which was first isolated from the bamboo rats (*Rhizomys sinensis*). *T. marneffei* was traditionally known for its high incidence and mortality rates in human immunodeficiency virus (HIV) patients. Epidemiological data reveal a concerning upward trend of infections among HIV-negative individuals, including immunocompetent hosts. At the meantime, although the bamboo rats have been reported to be associated with *T. marneffei* infection, there is a noticeable rising trend of the bamboo rats hunting and farming industry. Public awareness regarding the zoonotic transmission risks associated with these rodents remains limited.

**Case presentation:**

We report five cases of *T. marneffei* infection occurring within a single year, all involving individuals with a history of hunting wild bamboo rats (*Rhizomys* spp., likely *Rhizomys sinensis*). All five patients underwent HIV testing upon admission, with uniformly negative results. Notably, other immunodeficiency diseases, chronic comorbidities or prior immunosuppressive therapy were not found in these patients. The clustered emergence of these cases—affecting immunocompetent individuals within neighboring geographic areas over a brief timeframe, all sharing exposure through bamboo rat hunting—warrants detailed characterization. We herein present clinical profiles of these five cases.

**Conclusions:**

These cases demonstrate epidemiological associations between contact with wild bamboo rats and *T. marneffei* infections in immunocompetent individuals. The atypical clinical symptoms and variable imaging manifestations of *T. marneffei* infection may lead to increased underdiagnosis and misdiagnosis. Systematic implementation of exposure history, particularly documenting contact with wild animals for patients with pulmonary infection to make a timely diagnosis. This study also underscores the urgent need for public awareness regarding the potential risks of *T. marneffei* infections associated with hunting wild bamboo rats and the bamboo rat farming industry.

**Supplementary Information:**

The online version contains supplementary material available at 10.1186/s12879-025-10713-z.

## Introduction

*Talaromyces marneffei* (*T. marneffei*) is a thermally dimorphic fungus that can cause fatal systemic fungal diseases that are endemic to tropical and subtropical Asia [1]. *T. marneffei* infection is one of the most common opportunistic diseases among human immunodeficiency virus (HIV)-positive people living in endemic areas [2, 3]. However, in recent years, with advances in cancer treatment, organ transplantation, and the use of immunosuppressants, the number of infections in HIV-negative individuals has been increasing rapidly. *T. marneffei* infection in HIV-negative individuals often poses diagnostic challenges and may be easily overlooked. Therefore, clinicians should prioritize increased attention to *T. marneffei* infection in HIV-negative patients [1, 4].

Bamboo rats (*Rhizomys* spp.) are among the few mammals that subsist on a bamboo-based diet and are widely farmed in southern Chinese provinces as a unique meat animal. Decades ago, there were more than ten thousand farms [5]. However, there have been numerous reports identifying bamboo rats as a reservoir of that opportunistic pathogen [6, 7]. *T. marneffei* was first isolated from the organs of bamboo rats (*Rhizomys sinensis*) in 1956, and occasional clinical reports of *T. marneffei* infections associated with bamboo rats have been published since then [8]. Nevertheless, comprehensive reports on this matter remain scarce. Given the increasing trend of the bamboo rats hunting and farming industry, it is crucial to underscore the importance of raising public awareness regarding the risks associated with bamboo rats. Our team treated five patients infected with *T. marneffei* from June 2023 to June 2024. All patients exhibited pulmonary lesions or respiratory symptoms as their initial presentation. Interestingly, all of these five cases were occurred in otherwise healthy individuals without underlying illness who hunted wild bamboo rats (*Rhizomys spp*, probably *Rhizomys sinensis* considering that all these patients hunted in provinces located in southern Chinese provinces, though specific detail were not available) prior to admission to the hospital. All patients received timely antifungal therapy, leading to improvement in their conditions.

In this study, we report five cases of pneumonia caused by *T. marneffei* infection. These cases provide valuable reference for the diagnosis and treatment of *T. marneffei* infection and underscore the importance of raising medical awareness regarding contact with bamboo rats.

## Methods

We conducted a retrospective analysis of five *T. marneffei* infection patients without underlying illnesses admitted to Zhejiang Provincial People’s Hospital between June 2023 and June 2024. All patients had a history of close contact with wild bamboo rats prior to the onset of their illness. The inclusion and exclusion criteria for this study were as follows: Inclusion criteria: (1) Patients with confirmed *T. marneffei* infection. (2) Patients with a history of hunting or close contact with wild bamboo rats. Exclusion criteria: (1) Patients with immunodeficiency diseases or undergoing immunosuppressive therapy. (2) Patients who had undergone organ transplantation. (3) Patients with a history of chronic underlying diseases. All data were collected using a standardized form based entirely on the medical records of each patient. We gathered and analyzed the following information: medical history, clinical manifestations, laboratory indexes, medical imaging findings, complications, and prognosis.

This study protocol was conducted in accordance with the Declaration of Helsinki and approved by the Ethics Committee of Zhejiang Provincial People’s Hospital. We obtained all written informed consent from the five patients for the publication of any potentially identifiable images or data included in this article.

## Case reports

### Case-patient 1

On June 26, 2023, a 55-year-old woman was admitted to the hospital with a chief complaint of coughing and sputum for half a month. The patient reported the onset of cough and sputum, accompanied by sore throat, chest tightness, and shortness of breath, 15 days prior to admission. In addition, the patient also experienced intermittent abdominal distension and nausea, but without vomiting or fever. She consulted a pulmonologist and was admitted to the hospital. Physical examination revealed coarse breath sounds. Laboratory findings showed that inflammatory markers, including C-reactive protein (CRP), white blood cell (WBC) count, and neutrophil count, were within normal limits. Galactomannan and β-D-glucan tests were negative. Moreover, lymphocyte subpopulation analysis indicated that the absolute counts of lymphocytes, CD3^+^ T cells, CD19^+^ T cells, and natural killer (NK) cells were decreased, while B cells remained within the normal range. Other laboratory indexes are shown in Table 1.

**Table 1:** Clinical laboratory indexes of five patients

Parameter	Patient 1	Patient 2	Patient 3	Patient 4	Patient 5	Reference value
WBC (10^9^/L)	3.92	8.07	4.07	4.80	6.64	3.5–9.5
Neutrophil (10^9^/L)	2.14	5.51	1.63	2.45	3.71	1.8–6.3
lymphocyte (10^9^/L)	0.92	1.55	1.79	1.94	2.10	1.1–3.2
CRP (mg/L)	8.40	1.30	1.80	1.50	6.00	<10
Procalcitonin (ng/ml)	0.04	0.05	0.04	0.04	0.02	<0.25
CD3^+^CD4 ^+^ (10^6^/L)	445	-	1037	-	795	537–1282
CD3 ^+^ CD8^+^ (10^6^/L)	171	-	254	-	143	258–1042
Natural killer cell (10^6^/L)	8	-	178	-	925	44–436
Latency periods	3 weeks	4 weeks	4 weeks	2 weeks	4 weeks	-
Diagnostic methods	Tissue biopsy, etiologic culture, mNGS	Tissue biopsy, diagnostic therapy	Tissue biopsy, mNGS	Tissue biopsy, mNGS, diagnostic therapy	Tissue biopsy, etiologic culture, mNGS	-
CT results	Diffusely distributed multiple patchy shadows in both lungs	An irregular mass-like area of increased density in the right upper lobe	Multiple nodular shadows in both lungs	Scattered patchy inflammato-ry lesions in both lungs	A mass in the right middle lobe of the lung	-
Fever	no fever	no fever	no fever	no fever	no fever	-

mNGS: Metagenomic Next-Generation Sequencing

**Fig. 1:**
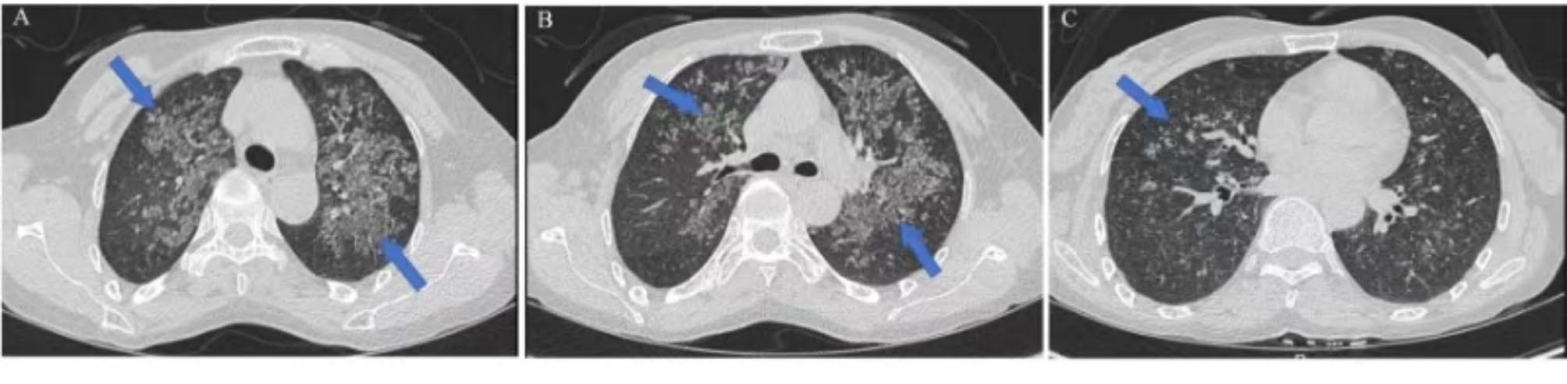
Chest CT scan of patient 1. **A-C.** The different layers of chest CT images at the time of patient 1 show diffusely distributed multiple patchy shadows in both lungs with unclear boundaries (blue arrow)

The CT scan of the patient’s lungs at admission revealed a diffuse distribution of multiple patchy shadows in both lungs, indicating a high possibility of infectious lesions in both lungs, and enlarged lymph nodes under the armpit (Fig. 1).

**Fig. 2:**
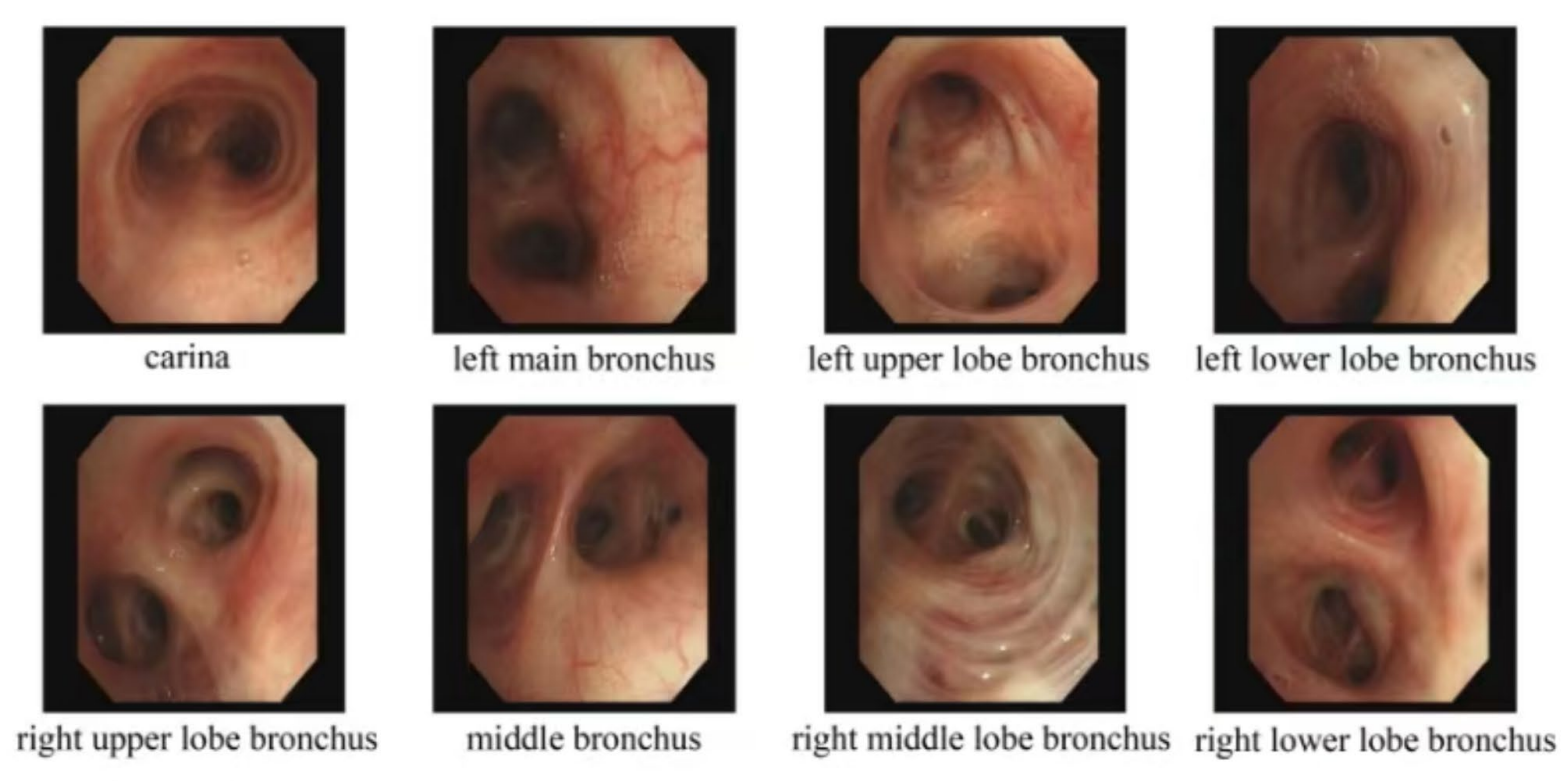
Bronchoscopy of the patient 1. Bronchoscopy reveals that all lumens are patent, with no obvious neoplasms observed. Lavage and brush cytology were performed on the apical segment of the right upper lobe

Systemic positron emission computed tomography examination also revealed multiple instances of lung inflammation and lymph node enlargement, including in the neck, bilateral hilar, mediastinum, and right axilla. Upon admission, a detailed review of the patient’s medical history revealed a significant history of hunting wild bamboo rats approximately 3 weeks prior to the onset of her illness. During her hospitalization, after confirming the absence of contraindications, a bronchoscopy was performed, which revealed a clear airway with no evidence of neoplastic lesions (Fig. 2).

**Fig. 3:**
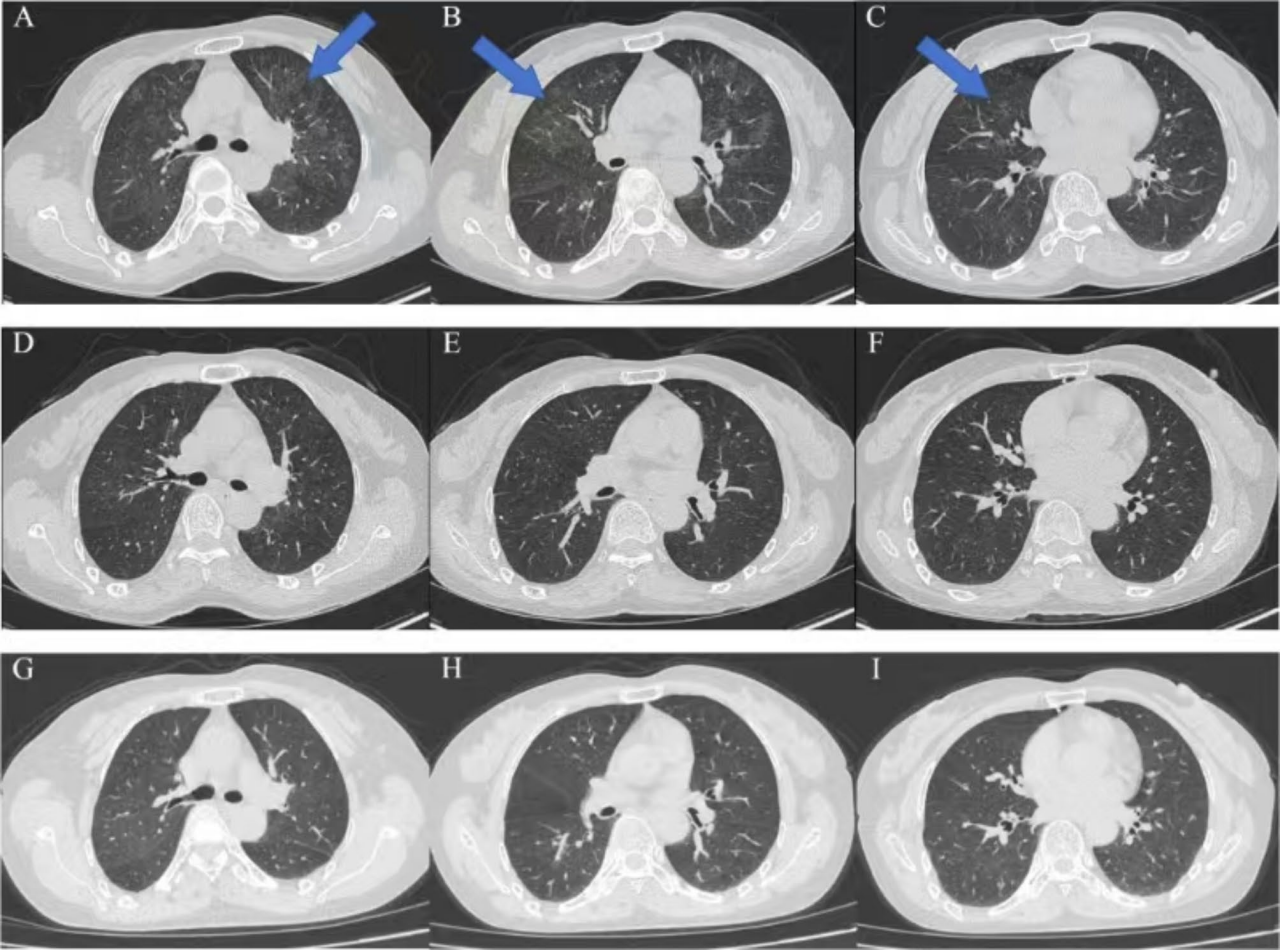
The dynamic changes in chest CT imaging of patient 1 during three follow-up examinations after discharge. **A-C.** The chest CT scan taken on the 34th day after the diagnosis of *T. marneffei* infection. The images reveal multiple diffuse ground-glass opacities in both lungs with unclear boundaries. Compared to previous scans, the extent of the lesions has decreased (blue arrows). **D-I.** Figure D-F and G-I represent the chest CT scans taken on the 88th and 180th days, respectively, after the diagnosis of *T. marneffei* infection. These images show minor infectious lesions in both lungs, with significant absorption compared to the earlier chest CT

**Fig. 4:**
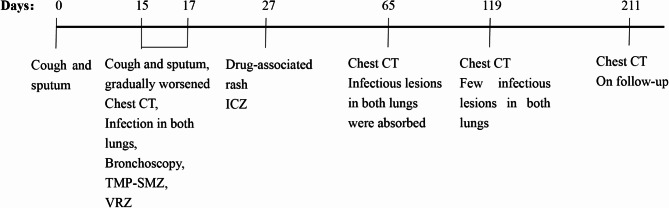
Timeline of medical history of patient 1. A brief overview of patient 1 from disease onset (day 0) to the fourth chest CT scan (day 211) is shown in the time follow-up chart. TMP-SMZ: trimethoprim-sulfamethoxazole, VRZ: voriconazole, ICZ: itraconazole

Bronchoalveolar lavage fluid (BALF) metagenomic next-generation sequencing (mNGS) identified *Talaromyces marneffei* (*T. marneffei*) and *Pneumocystis jirovecii* infection. The tuberculosis (TB) -related tests (including the nuclei acid of mycobacterium TB, acid-fast staining and T-SPOT tests) were negative. The patient was treated with voriconazole and the compound sulfamethoxazole (trimethoprim-sulfamethoxazole). In this case, the infection was suspected to be linked to the patient’s consumption of bamboo rats, which have been previously reported as hosts of *T. marneffe*i [9]. Following 10 days of treatment with voriconazole and the compound sulfamethoxazole, the patient developed scattered erythematous lesions on the trunk accompanied by pruritus, which progressively worsened and coalesced into larger plaques. After a dermatological consultation, the rash was considered to be related to the usage of compound sulfamethoxazole. Consequently, we discontinued this agent and switched to itraconazole as monotherapy, leading to gradual resolution of the rash. Etiological culture of alveolar lavage fluid on July 12, 2023, confirmed *T. marneffei* infection. The patient exhibited multiple enlarged lymph nodes, and a needle biopsy specimen of the left submandibular lymph node on July 10, 2023 was positive for *T. marneffei*. After a comprehensive assessment of the patient’s clinical history and examination findings, the patient was discharged. At 34, 88, and 180 days after BALF culture confirmed *T. marneffei* infection, the patient underwent chest CT examinations at our outpatient department. These imaging studies demonstrated progressive absorption of the pulmonary infectious lesions, with only a few residual foci observed (Fig. 3). The patient is now undergoing regular follow-up at the clinic. The medical timeline is presented in Fig. 4.

### Case-patient 2

**Fig. 5:**
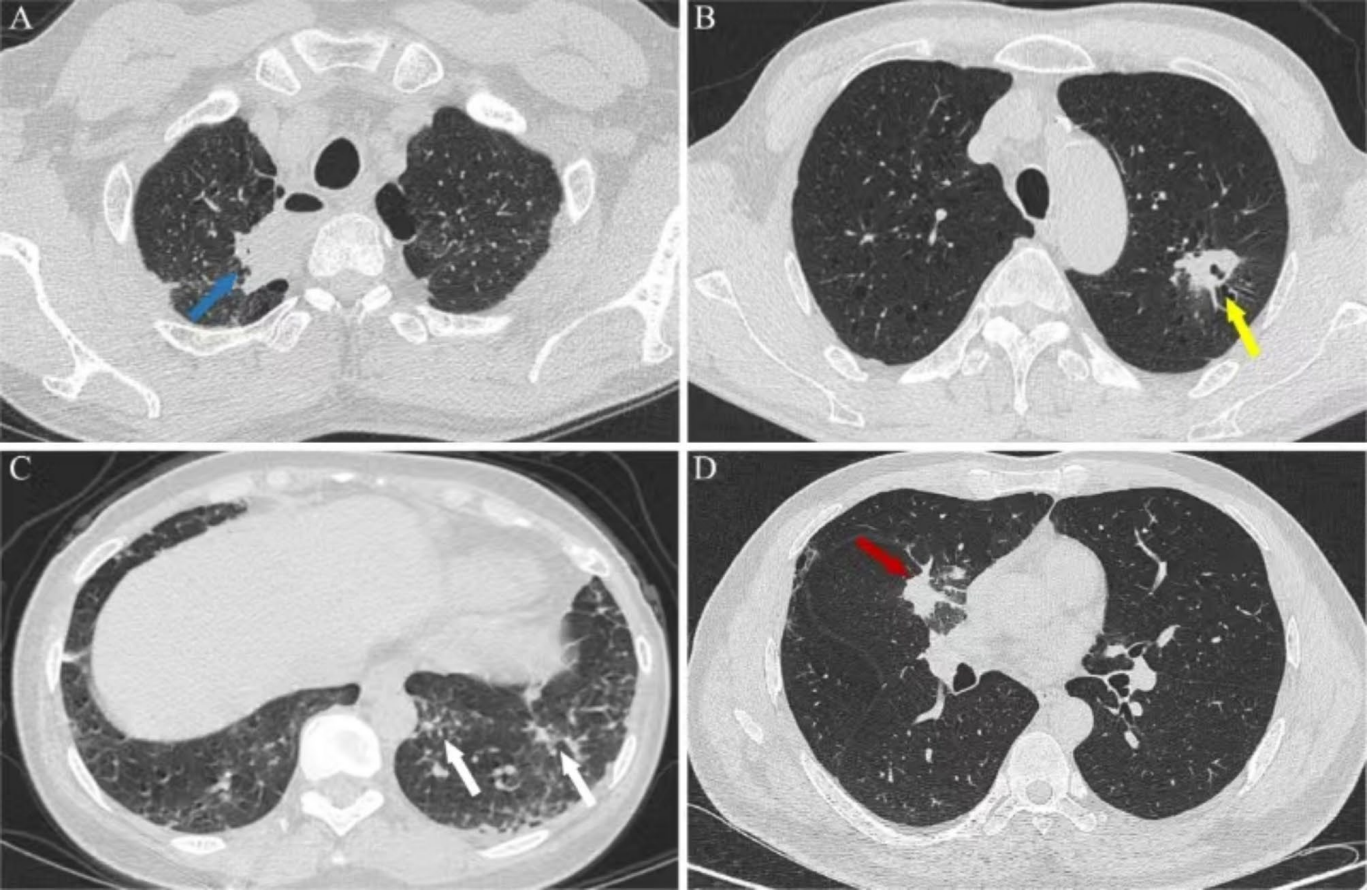
The chest CT images of pulmonary infection caused by *T. marneffei* exhibit a variety of features. **(A)** The chest CT of patient 2 shows a lesion in the right upper lobe of the lung, with an irregular mass-like area of increased density in the right upper lobe (blue arrow). **(B)** The chest CT of patient 3 shows multiple nodular shadows in both lungs, with some nodules in the left lung exhibiting cavitation (yellow arrow). **(C)** The chest CT of patient 4 shows scattered patchy inflammatory lesions in both lungs (white arrow). **(D)** The chest CT of patient 5 shows a mass in the right middle lobe of the lung, with poorly defined borders (red arrow)

The second patient, a 59-year-old male, was admitted to the hospital on January 4, 2024, with a cough, sputum and chest distress for 10 days without accompanying fever. Laboratory findings showed normal levels of CRP, WBC and neutrophil count (Table 1). Both galactomannan and β-D-glucan tests were negative. Chest CT revealed lesions in the upper lobe of the right lung (Fig. 5A).

On the second day after admission, a bronchoscopy examination was performed, which revealed local mucosal carbon pigmentation in the upper and middle lobes of the right lung, with no abnormalities found in the rest of the lung. Furthermore, mNGS and bacterial culture of BALF from this patient did not reveal any abnormalities, and the TB-related tests were negative. The patient was subsequently treated with piperacillin and tazobactam. After one week of treatment, chest CT imaging did not show significant absorption of the lesions. We then carefully examined the medical history and found that approximately one month prior to the onset of symptoms, the patient had captured and consumed wild bamboo rats. Therefore, a CT-guided percutaneous lung biopsy was arranged, and the etiologic culture result was negative. However, histopathological examination of the biopsy specimen from the right upper lobe nodules showed chronic granulomatous inflammation with increased eosinophilic infiltration, suggesting a suspected infection with *T. marneffei*. After discussing treatment options with the patient, it was decided to initiate voriconazole diagnostic antifungal therapy. Subsequent CT images demonstrated improved lesions. The patient is currently undergoing regular follow-up at the outpatient department.

### Case-patient 3

The third patient was a 56-year-old healthy male with no prominent symptoms of cough, sputum or fever. Due to the discovery of enlarged pulmonary nodes, he went to the pulmonary specialist clinic on November 5, 2023 and reported a history of hunting and consuming bamboo rats approximately 1 month prior to the visit. A chest CT imaging revealed multiple pulmonary nodules bilaterally, with findings initially suggestive of granulomatous lesions (Fig. 5B). Laboratory investigations showed normal levels of CRP, WBC and neutrophil count (Table 1). Both galactomannan and β-D-glucan tests were negative. After admission, the patient was given bronchoscopy examination, and the TB-related tests were negative. In order to confirm the diagnosis, the patient underwent a percutaneous puncture biopsy of pulmonary nodules. Histopathological examination of the lung biopsy revealed chronic granulomatous lesions with coagulative necrosis. Metagenomic examination of the lung tissue mass confirmed an infection with *T. marneffei*. However, after 5 days of liposomal amphotericin B treatment, he developed significant chills, leukopenia, abnormal liver function, and elevated brain natriuretic peptide levels. Due to the adverse effects associated with liposomal amphotericin B, voriconazole antifungal therapy was initiated instead, along with supportive treatments for liver protection. The patient is currently being regularly followed up in the outpatient department.

### Case-patient 4

The fourth patient, a 55-year-old woman, was admitted to the hospital on January 4, 2024 due to a 2-week history of cough and dyspnea on exertion. The patient presented with cough, dyspnea after exertion, no sputum, and no fever. Hematological examination at a local hospital revealed slightly elevated CRP. The patient reported a history of hunting and consuming wild bamboo rats approximately 2 weeks prior to the onset of symptoms. The patient subsequently admitted to our hospital for further evaluation and treatment. After admission and comprehensive laboratory investigations, the CRP, WBC, and neutrophil counts were within the normal range (Table 1), and the chest CT revealed multiple inflammatory lesions in both lungs (Fig. 5C). The galactomannan testing and β-D-glucan testing were negative. A bronchoscopy examination was conducted, and no significant abnormalities were found in the visual range of the bronchoscope. We performed lavage on the left lower lobe bronchus and tested the BALF. The mNGS of the BALF revealed *T. marneffei* infection, and the TB-related tests were negative. The patient was initiated on empirical voriconazole antifungal therapy and the chest CT image showed the lesions were improved. She is currently undergoing regular follow-up at the outpatient department.

### Case-patient 5

The fifth patient, a 46-year-old male, was admitted to the hospital on May 4, 2024, following the discovery of a pulmonary shadow during a routine health examination. The patient reported symptoms of cough, no sputum, and no fever. Laboratory investigations showed normal levels of CRP, WBC and neutrophil counts (Table 1). The galactomannan testing and β-D-glucan testing were negative. Enhanced chest CT revealed images of the right middle lobe of the lung with local bronchial stenosis and multiple enlarged lymph nodes in the right hilar and mediastinal regions (Fig. 5D). A bronchoscopy examination was conducted, and new organisms were observed in the subbranch of the right middle lobe. Biopsy of the lung lesion under bronchoscopy was performed, and histopathological examination revealed chronic granulomatous inflammation, primarily suggestive of a fungal infection. The TB-related tests were negative. Metagenomic sequencing of the biopsied lung tissue confirmed *T. marneffei* infection. Interestingly, sputum etiological testing also identified *T. marneffei* infection. A detailed review of the patient’s medical history revealed that he had hunted and consumed wild bamboo rats approximately 1 month prior to the onset of cough. The patient was initiated on voriconazole antifungal therapy and is currently undergoing regular follow-up at the outpatient department.

## Discussion

*T. marneffei* was first isolated in 1956 from the livers of wild bamboo rats [8, 10]. Bamboo rats and their burrow soils serve as important reservoirs for *T. marneffei*. Identification and typing of the fungus demonstrated that several isolated from bamboo rats and humans shared the same multi-locus genotype [11, 12]. *T. marneffei* infection significantly threatens populations in endemic regions, especially HIV-positive individuals who face a heightened risk [13]. In recent years, the incidence of *T. marneffei* infection has also risen among HIV-negative patients, likely due to the growing number of organ transplantations, autoimmune diseases, and primary or secondary immunodeficiency conditions [14, 15]. However, the reports of healthy individuals infected with *T. marneffei* were not common. Our findings indicate that *T. marneffei* infection can occur in healthy individuals following exposure to wild bamboo rats. Public awareness of the potential risks associated with bamboo rats as carriers of pathogens, remains insufficient. The health risks posed by contact bamboo rats have been underestimated by the general population.

Previous studies have confirmed that CD4^+^ T cells are involved in the process of clearing *T. marneffei* infection in animals [16]. In humans, CD4^+^ T-cell-dependent immune deficiency has also been shown to be associated with fatal disseminated *T. marneffei* infections in HIV-positive patients, as well as a reduction in lymphocyte numbers and function in HIV-negative patients with *T. marneffei* infections [17, 18]. Furthermore, T-cell abnormalities have been observed post-infection, indicating that the incidence of *T. marneffei* infection is not solely dependent on pre-existing immune deficiencies. *T. marneffei* infection is capable of compromising the immune barrier in healthy individuals following exposure to wild bamboo rats [18].

An increasing number of *T. marneffei* infections have demonstrated that HIV-negative *T. marneffei* infections exhibit higher mortality rates compared to HIV-positive cases [19, 20]. This phenomenon may be attributed to delayed diagnosis due to a lack of clinical suspicion. *T. marneffei* infections typically present with atypical symptoms and diverse imaging findings, as observed in our reported cases. *T. marneffei* infection may be misdiagnosed as TB due to its atypical clinical manifestations and varied imaging features, leading to delayed treatment initiation [21, 22]. Receiving long-term anti-TB treatment for these misdiagnosed patients can result in additional complications and elevated mortality [23, 24]. Clinicians should remain vigilant regarding the evolving epidemiology, particularly in HIV-negative patients [21]. In our study, five patients infected with *T. marneffei* had normal immune function and no other chronic diseases before onset. Previous studies have emphasized the role of underlying diseases in cases related to *T. marneffei* infection, but excessive focus on such factors may lead to missed diagnosis and misdiagnosis of *T. marneffei*. Compared to traditional histopathologic examinations, highly sensitive diagnostic techniques offer effective alternatives in clinical practice. Immunobinding tests such as galactomannan and β-D-glucan tests can rapidly indicate the presence of invasive fungal infections [25]. Polymerase chain reaction (PCR)-based methods such as mNGS can sensitively detect a large spectrum of pathogens [26]. Although PCR-based methods carry a risk of false-positive results, they are valuable for identifying potential pathogens and initiating early treatment, particularly when histopathologic results are inconclusive. Early diagnosis, along with timely antifungal therapy, are keys to a favorable prognosis.

*T. marneffei* infections can affect the skin, respiratory system, digestive system, and lymph nodes, resulting in either localized or diffuse infections [3, 27, 28]. In addition, previous studies have suggested that the respiratory system may be the most frequently and initially affected organ [24]. The imaging findings of lung involvement in *T. marneffei* infections are highly variable, and no two of the five patients described in our study exhibited identical radiographic features. Therefore, in clinical practice, it is particularly important to carefully inquire about the patient’s history of the disease. If there is a confirmed history of contact with wild bamboo rats, *T. marneffei* infection should be considered as a potential diagnosis. Clinicians should provide health education to patients to enhance their awareness of epidemic prevention related to wild bamboo rats. Furthermore, patients infected with *T. marneffei* in these cases were all associated with wild bamboo rats hunting, which poses significantly higher pathogenic risks compared to farmed bamboo rats. To mitigate further epidemic risks, measures such as discouraging the hunting of wild bamboo rats are necessary.

Early diagnosis and effective antifungal therapy are essential to improve prognosis [29]. The first-line treatment for *T. marneffei* infection is amphotericin B, while voriconazole and itraconazole are equally effective. Blood levels of voriconazole must be carefully monitored during treatment [3]. Patients who do not receive appropriate antifungal therapy have a poor prognosis. Voriconazole is an effective and well-tolerated treatment option for diffuse *T. marneffei* infection [30, 31]. Studies have shown that amphotericin B and itraconazole are the primary strategies for treating *T. marneffei*. Compared to itraconazole, amphotericin B treatment can significantly accelerate the fungal clearance rate and reduce the recurrence rate. However, amphotericin B has a relatively high incidence of side effects [32]. The newly developed liposomal amphotericin B provides a safer alternative but it is currently much more expensive than itraconazole or voriconazole which limited its prevalence [33]. Itraconazole or voriconazole are well-tolerated and are widely used as alternatives to amphotericin B [34, 35]. If patients with *T. marneffei* infection receive timely antifungal therapy, the mortality is significantly reduced.

## Conclusion

Previous reports have widely identified the susceptibility to *T. marneffei* infection in individuals with immunodeficient conditions, which has practical implications for diagnosis. However, our study revealed that healthy individuals who hunted wild bamboo rats could also be infected with *T. marneffei*. On the one hand, clinicians should be aware that *T. marneffei* infection can occur in individuals without underlying diseases, which may be helpful for avoiding missed diagnoses or misdiagnoses. On the other hand, the increasing incidence of bamboo rat-associated *T. marneffei* infection in non-HIV patients is not only a medical problem, but also a multi-faceted challenge for public health, animal farming and social management. To effectively reduce the risk of *T. marneffei* infection associated with bamboo rats, it is necessary to strengthen public health education and awareness to discourage hunting wild bamboo rats, improve bamboo rat farming management and establish disease surveillance.

## Electronic supplementary material

Below is the link to the electronic supplementary material.

Supplementary Material 1

## Data Availability

The original contributions presented in the study are included in the article, and further inquiries can be directed to the corresponding author.

## Abbreviations

HIV: Human immunodeficiency virus
CRP: C-reactive protein
WBC: White blood cell
NK: Natural killer
BALF: Bronchoalveolar lavage fluid
NGS: Next generation sequencing
TB: Tuberculosis
