# Supplementary material for: Talaromyces Marneffei infections in five human immunodeficiency virus-negative patients with the history of predation on wild bamboo rats (Rhizomys spp) - a case series
Source: BMC Infect Dis. 2025 Mar 11;25:340. doi: 10.1186/s12879-025-10713-z (PMC11895350; doi:10.1186/s12879-025-10713-z)
Supplement: Supplementary file 1 — Supplementary Material 1 [file 12879_2025_10713_MOESM1_ESM.pdf]

CARE Checklist of information to include when writing a case report

CARE case report guidelines

(cc) BY-NC-ND

Topic	Item	Checklist item description	Reported on Line
Title	1	The diagnosis or intervention of primary focus followed by the words "case report"	Line1-2
Key Words	2	2 to 5 key words that identify diagnoses or interventions in this case report, including "case report"	Line 51
Abstract (no references)	3a	Introduction: What is unique about this case and what does it add to the scientific literature?	Line 37-39、47-50
	3b	Main symptoms and/or important clinical findings	Line 43-45
	3c	The main diagnoses, therapeutic interventions, and outcomes	Line 37-38
	3d	Conclusion—What is the main "take-away" lesson(s) from this case?	Line47-50
Introduction	4	One or two paragraphs summarizing why this case is unique (**may include references**)	Line 62-69
Patient Information	5a	De-identified patient specific information.	Line 75-76
	5b	Primary concerns and symptoms of the patient.	Line 76-78
	5c	Medical, family, and psycho-social history including relevant genetic information	Line 66-68
	5d	Relevant past interventions with outcomes	Line 98-100、110-112
Clinical Findings	6	Describe significant physical examination (PE) and important clinical findings.	Line 80-85、88-90
Timeline	7	Historical and current information from this episode of care organized as a timeline	Line75-77、112-113、117-120
Diagnostic Assessment	8a	Diagnostic testing (such as PE, laboratory testing, imaging, surveys).	Line 88-101
	8b	Diagnostic challenges (such as access to testing, financial, or cultural)	Line98-101,107-109.
	8c	Diagnosis (including other diagnoses considered)	Line107-109、116-118
	8d	Prognosis (such as staging in oncology) where applicable	N/A
Therapeutic Intervention	9a	Types of therapeutic intervention (such as pharmacologic, surgical, preventive, self-care)	Line 112-113
	9b	Administration of therapeutic intervention (such as dosage, strength, duration)	Line 112-113
	9c	Changes in therapeutic intervention (with rationale)	Line 114-119
Follow-up and Outcomes	10a	Clinician and patient-assessed outcomes (if available)	Line 117-118
	10b	Important follow-up diagnostic and other test results	Line119-122
	10c	Intervention adherence and tolerability (How was this assessed?)	Line 122-123
	10d	Adverse and unanticipated events	N/A
Discussion	11a	A scientific discussion of the strengths AND limitations associated with this case report	Line 217-221
	11b	Discussion of the relevant medical literature **with references**.	Line208-211、214-217...
	11c	The scientific rationale for any conclusions (including assessment of possible causes)	Line 214-219、248-252
	11d	The primary "take-away" lessons of this case report (without references) in a one paragraph conclusion	Line 272-280
Patient Perspective	12	The patient should share their perspective in one to two paragraphs on the treatment(s) they received	N/A
Informed Consent	13	Did the patient give informed consent? Please provide if requested	Yes ☐ No ☑
